# The Correction of Mal-Occlusion in Artificial Dentures*Read before the National Dental Association at its Twenty-third Annual Session, New Orleans, La., October 20-24, 1919, and reprinted from *Journal N. D. A.*

**Published:** 1920-09

**Authors:** M. M. House

**Affiliations:** Indianapolis, Ind.


### THE CORRECTION OF MAL-OCCLUSION IN ARTIFICIAL DENTURES *

BY M. M. HOUSE, D.D.S., INDIANAPOLIS, IND.

The primary meaning of the word "mal-occlusion" offers us but a rough approximation to a clear understanding of its significance in relation to artificial dentures. It comes from two Latin words which, together, signify a bad closing; in this case of course a bad closing of the teeth. What we have to consider, however, are a number of minute and subtle mechanical inaccuracies universally present in artificial dentures and what steps may be taken to avoid them. These mechanical inaccu-

* Read before the National Dental Association at its Twenty-third Annual Session, New Orleans, La., October 20-24, 1919, and reprinted from *Journal N. D. A.*

racies may be due to a variety of causes, chief among them being an undeveloped technic and the use of materials and appliances designed for ease and speed of production rather than for efficiency of function.

The starting point for every discussion of this subject, and the thought which underlies and summarizes the entire paper herewith submitted, is the broad and apparent startling principle that *all artificial dentures when first inserted in the mouth are mechanically imperfect.* This principle is so fundamentally important that it can scarcely be sufficiently emphasized or too often repeated. It is practically impossible to prepare an artificial denture which will not, on its first insertion, prove mechanically inaccurate to the point of involving mal-occlusion. This mal-occlusion, therefore, should be *corrected* since it can not be *prevented.*

A. The first step in the construction of dentures is a careful examination of the mouth. This must of necessity be a digital examination. The height of the tissue attachments around the labial and buccal borders of the upper mouth should be determined by placing the finger on the inside of cheek and lip, pressing upward and then outward and downward, observing the height of the attachments, their freedom of movement and the resistance of both muscle attachments and tissues. The buccal and labial borders of the lower mouth are examined in a similar manner. To test the tissues around the lingual border of the lower mouth anterior to the region of the first molars, have the patient place the tongue straight upward to the vault of the upper mouth, then observe with the eye the lingual muscle attachment, whether high or low as compared to the crest of the ridge, also whether it is a broad or a narrow attachment. With the forefinger test the height of tissue attachments around the region observed.

To examine the height and resistance of tissue attachments posterior to the region of the bicuspids, also the form and space below and posterior to the mylo-hyoid ridge, have the patient extend the tongue as far out of the mouth as possible and with the forefinger make careful and thorough examination. This gives a mental design from which to form an ideal tray in which to make an impression that will utilize all the space advantageous for the stabilizing and comfortable seating of the finished denture without in any way resisting Nature's laws.

Where the mouth is so formed that a flange is permissible either below or posterior to the mylo-hyoid ridge, it affords the greatest possible advantage for the permanent stabilizing of a lower denture.

The mouth form and tissue attachments in this region vary widely on either side, seldom being symmetrical. Hence it is of the utmost importance to make the careful examination above described and to form in one's mind an ideal design as a preliminary step in the procedure. Otherwise, the most successful results can not be obtained. There are cases which will not permit of a lingual flange on lower plates. Such a flange, therefore, should never be attempted in mouths in which it is contra-indicated.

It is necessary not only to note and understand tissue conditions in this thorough and detailed manner, it is equally necessary to note similarly the size and form of the mouth, as well as the size and form of the throat.

We may classify mouths, in respect to size, in three groups.

1. Those of large size, both above and below, affording the greatest possible advantage in the utilization of stabilizing influences.

2. Those of medium size and affording a somewhat lesser degree of such advantage.

3. Those so small as to present difficulties in stabilizing the dentures, destroying a large part of their efficiency for service.

This classification may have to be applied separately to upper and lower mouths, many persons having two distinct types; as, for instance, the prognathous or the retrusive lower, the former combining a Class Three upper with a Class One lower, while the latter gives us a Class One upper and a Class Three lower, in the *extreme* cases.

When we consider the physical form of the mouth we find:

1. The very symmetrical mouth, large or medium in size, with good vault and ridges, accompanied by a large or medium throat.

2. The medium size mouth with a high or medium vault, medium size throat and of equal symmetry, a mouth which can sometimes be improved by surgical interference.

3. The mouth of unequal symmetry, having perhaps a high, tortuous vault, thick ridges, heavy undercuts, large tuberosities, or perhaps with a flat vault, badly absorbed ridges, thin, sensitive tissue, and very hard.

The size and form of the throat tend to correspond with the mouth conditions and particularly with the size of the mouth. Class One mouths are usually accompanied by a large throat, with the immovable tissue of the soft palate extending posteriorly to the hard palate, from three-sixteenths to one-half inch, at which point the curtain of the soft palate becomes movable with a decidedly large range of movement. Class Two mouths are usually accompanied by throats of medium size, usually good in form, but with immovable tissues of the soft palate extending posteriorly to the hard palate proportionately less than in Class One. Class Three mouths are usually accompanied by throat forms proportionate in size with the mouth classifications, and with little or no immovable tissue posterior to the hard palate, the curtain of the soft palate turning down very abruptly at or within one-eighth inch of the junction of the hard and soft palates. These mouths are the most difficult upon which to construct stable and efficient dentures. They are usually devoid of tuberosities, thereby presenting difficulties in establishing proper posterior length and valve seal in this region without creating irritation and soreness.

Throat form, size and tissue conditions as regards immovable tissue and its distance posterior to the hard palate affect upper dentures only. No denture should be allowed to extend distally to the edge of the movable curtain of the soft palate, and in medium and large throats where the movable curtain is distal to the soft palate to any marked degree the plate should be constructed to extend posterior to the hard palate only to sufficient distance to give proper form to the posterior edge of the plate and allow for valve seal.

I have spoken of the mouth examination in such detail and at such length because I am convinced that without an exact preliminary conception of conditions to be met as determining the design, even the grosser mechanical imperfections of artificial dentures can scarcely be avoided. I now pass on to speak of the impression.

B. The impression offers a host of opportunities and loopholes for the ingress of inaccuracies. No method ever devised can guarantee the production of a cast over which a denture may be constructed which will compensate and allow for all the forms of stress to which it is subjected, or be capable of distributing them impartially over that portion of the mouth which is designed to sustain the forces and stresses involved in mastication. The changes which inevitably occur in the materials used for receiving the impression, such as expansion and contraction, disintegration due to moisture and to the processes of vulcanization, necessarily open many avenues to trouble, particularly when a technic is employed which is no better than the average.

The construction of casts for this work has been done in a manner generally lax and careless to a marked degree. The plaster of paris cast, as ordinarily used, is entirely unsuited for this purpose. The reason why this is so has been set forth at great length in Prothero's chapter on "Impressions and Impression Materials." (See particularly pages 52-57 of his Prosthetic Dentistry, revised edition.) It will suffice here to remind you that plaster expands during and after setting, giving rise to an unavoidable warpage, and a consequent distorted reproduction of the mouth it is intended to represent in such necessarily minute detail.

The best materials available is Spences plaster, or Cassalith, or some similar substance, and nothing poorer should be employed for making casts. But even a good material for the cast is no guarantee of success unless it is used as directed and recommended by the best and most careful technicians, with the greatest possible care exercised in its manipulation.

No single step in this work can properly be said to be of minor importance, but there are some which embody a greater relative danger of error than others. The subject of bite plates and their construction offers an opportunity for the exercise of the utmost skill, and demands, indeed, the most intelligent understanding. A bite plate that is not properly reinforced and adapted can not possibly perform its function. The materials employed should be the very best obtainable and the technic used the most exacting possible. And to this end a thorough knowledge of the oral cavity and all its parts is absolutely essential. The needed accuracy simply can not be realized unless the operator has a much better knowledge of dental anatomy than that ordinarily possessed.

C. In my opinion no step in the production of dentures requires more skill or presents greater opportunities for inaccuracy than the articulating co-ordination or the setting up of the teeth. Here is required not only a thorough knowledge of anatomical form and of the various angles of stress operating during mastication, but the operator must also be able to exercise, to a superlative degree, his sense of art. He must be able to distinguish the various typical forms and to effect a successful blending of them. The ability to restore facial contour and expression in a pleasing and harmonious manner is quite as necssary to the construction of successful dentures as is the execution of a well-developed technic.

In the first place, care must of course be taken when articulating the casts to place them in precisely the relative position recorded in the bite plates. The slightest variation here will affect the entire plan of the completed denture.

Next, the operator is confronted with a danger of inaccuracy in the material used; namely, porcelain. There are required at least three transfers from the original carvings of plaster teeth to make a metal mold. Then, too, it is very rare for a complete set of teeth to go through the baking process without having one or more of the teeth come out checked or warped. This requires a replacement of these imperfect teeth with others from a different baking. Now the porcelain, when pressed in molds and baked, shrinks about one-sixth in size in all dimensions, and teeth made from the same mold but baked for a slightly different length of time and at a slightly varying temperature will inevitably vary from one another in size and form. This fact alone is of sufficient importance to make correct occlusion and articulation impossible in every case where the cause referred to operates. The clinical result of using teeth embodying such inaccuracies as these here described may be summed up in the single word, "inefficiency." The reaction varies with the individual. In some cases the denture is thrown down, while in others the patients become scientific jugglers and develop the use of a "chop bite." But in no case do they are can they have the highest degree of masticating efficiency.

Only the ignorance of the human beings with whom we have to deal and the kindness and forbearance of Mother Nature, have made it possible for us, as a profession, to satisfy our patients measurably, despite the employment of methods so glaringly indifferent and inaccurate.

So far as I have been able to ascertain, the first members of our profession to approach a scientific solution of this problem here indicated were Dr. G. V. Black and the Green brothers. The former chewed emery powder to correct the inaccurate and faulty articulation in his plates, and the Green brothers used a spring to open an old plain line articulator in which they had arranged for lateral movements, and then they placed it under a cam-shaped wheel on the lathe. They would then feed the carborundum on the teeth to ''chop the bumps off,'' as they pithily described the process.

But the most accurate, convenient and scientific method for eliminating these inevitable inaccuracies has been given us by Dr. Rupert E. Hall, of Chicago. The following is a concise description of his process:

After the case has been set up, tried in the mouth, waxed up, flasked, packed, vulcanized and finished, all of which must have been done with the utmost care and precision, the upper denture is placed in the mouth. The occlusal surfaces and incisal edges of the teeth on the lower denture are next covered with base plate wax and immersed in hot water of sufficient temperature to soften the wax thoroughly. They are then placed in the mouth and the patient is instructed at the same time to close, thereby giving correct occlusion. The dentures are then removed from the mouth, the wax is chilled, the dentures replaced together and firmly sealed in that relation. They are then remounted by the use of the bite jig on the Hall anatomical articulator, to be ground to the proper occlusion and articulation with carborundum powder No. 120, made into a paste with surgeon's lubricating jelly.

The advantage of Dr. Hall's method for correcting the inaccuracies that lead to mal-occlusion, as given above, are to be found in the systematization of the technic and in the designing of an articulator that is rigid and definite in its movements and possibilities of control, thus eliminating inaccuracies.

